# Racial/Ethnic Differences in Physiological Stress and Relapse among Treatment Seeking Tobacco Smokers

**DOI:** 10.3390/ijerph16173090

**Published:** 2019-08-25

**Authors:** Monica Webb Hooper

**Affiliations:** Case Comprehensive Cancer Center, Case Western Reserve University Cleveland, OH 44106, USA

**Keywords:** tobacco smoking, stress, cortisol, hypothalamic-pituitary-adrenal axis, racial differences, smoking relapse, African Americans

## Abstract

Stress is robustly associated with tobacco smoking and relapse. African Americans experience greater difficulty quitting compared to whites, yet no studies have examined race differences in physiological stress biomarkers during a quit attempt. This pilot study compared cortisol levels among treatment-seeking African American and white smokers, and relapse rates. Adult smokers (N = 115; n = 72 African American, n = 43 White) received eight sessions of group cognitive behavioral therapy plus transdermal nicotine patches. Assessments included demographics, salivary cortisol (collected at session 1, the end-of-therapy [EOT], and one-month post-therapy), and carbon monoxide-verified smoking relapse. Overall, cortisol levels declined over the course of the day at baseline, the EOT, and the one-month follow-up. African Americans exhibited lower cortisol levels compared to Whites at baseline and the EOT, but not at the one-month follow-up. In addition, African American smokers exhibited flatter slopes compared to Whites at each time point. Relapse rates were greater among African Americans at the EOT and one-month follow-up. The attenuated cortisol pattern observed in African Americans may indicate hypothalamic-pituitary-adrenal axis (HPA) exhaustion and aid our understanding of tobacco-related disparities. There is a need to focus on stress mechanisms and specific intervention approaches in order to eliminate racial/ethnic differences.

## 1. Introduction

Understanding and addressing tobacco-related health disparities is important for improving population health. Tobacco-associated diseases, such as cancer, stroke, cardiovascular disease, hypertension, and diabetes are disproportionately prevalent among African Americans compared to whites [1,2]. Racial/ethnic minorities are also less likely to quit smoking and more likely to relapse compared to their white counterparts [1,3,4,5]. Psychosocial factors such as perceived stress are associated with both smoking [6,7] and tobacco-associated medical illnesses [8,9]. The elevated levels of stress in African Americans [10] may manifest in greater difficulty quitting and subsequent health problems. However, the extant literature has not examined racial differences in physiological stress among tobacco smokers.

Robust associations exist between current tobacco smoking and perceived stress, such that smokers report greater stress compared to nonsmokers [6,11,12,13]. Multiple types of stress, such as finances, relationship conflicts, and discrimination are positively related to persistent smoking [14,15] and inversely associated with cessation [14]. Racial/ethnic minorities are underrepresented in the stress and smoking literature despite their overrepresentation in populations with chronic and unique stressors (e.g., low socioeconomic groups) [16,17,18]. Moreover, racial group is associated with both recent and lifetime stressful events, irrespective of socioeconomic status [19]. The extant data demonstrates that the positive associations between current smoking and perceived stress generalize to African Americans [11,12,13]. In addition, previous studies have found inverse associations between perceived stress and smoking cessation among African Americans [20,21].

The hypothalamic-pituitary-adrenocortical (HPA) axis is one of the neurobiological systems that directly regulates the physiological stress response [22,23]. Cortisol is the most commonly measured peripheral biomarker of stress. Upon exposure to a stressor, the HPA axis releases corticotropic releasing factor (CRF) [24], which promotes secretion of glucocorticoids from the anterior pituitary, such as cortisol, into circulation. In addition to responding to immediate stressors, cortisol concentration follows a diurnal rhythm, peaking just after waking and declining gradually over the course of the day [25]. 

According to the allostasis model [26], normal patterns of HPA axis regulation can be disrupted by repeated exposure to stressors. Repeated activation of the HPA axis due to environmental challenges may result in dysregulation of the stress response system, which is a risk factor for illness and disease [27]. Another way that normal patterns can be disrupted is through chronic nicotine exposure, which contributes to elevated cortisol levels among smokers [28,29]. Such prolonged exposure may result in enduring changes of the dopaminergic, cholinergic, and opioidergic systems [30]. In addition, HPA activity in response to stress regulates dopaminergic reinforcement of stimulants, such as nicotine [31]. For example, previous research found that attenuated cortisol responses to stress predicted relapse within a month among smokers who were abstinent for at least 24 hours at baseline [32].

Absent from the literature is research examining racial/ethnic differences in physiological stress among tobacco smokers. The allostasis model suggests that greater exposure to stress may contribute to racial/ethnic health disparities [26] mediated by the physiological stress response. The extant literature provides support for this model, demonstrating differential HPA responding by race/ethnicity. Following a public speaking stressor, previous research has found greater cortisol secretions among white versus African American men and women [33]. Other work, has found greater perceived stress among African Americans relative to whites, and higher cortisol upon awakening and flatter slopes over the day [26]. This blunting pattern in cortisol slopes may be a result of chronic stress exposures that lead to hormonal dysregulation. Other research has found that African Americans who reported high cumulative stress (e.g., interpersonal violence, discrimination, negative family-related life events, and community violence) exhibited lower awakening cortisol levels and a flatter slope throughout the day compared to Hispanics [34]. The greater allostatic load (i.e., the cumulative physiological toll of chronic stress on the body) may lead to HPA exhaustion among African Americans [35], and hence aid our understanding of smoking cessation challenges in this population.

Cognitive behavioral interventions for tobacco cessation have demonstrated efficacy in racially and ethnically diverse samples [36], and among African Americans [37]. This approach includes a focus on stress management, particularly for coping with nicotine withdrawal. Compared to whites, treatment seeking African American smokers report greater perceived stress at baseline and a greater reduction in stress following cognitive behavioral therapy (CBT) for tobacco cessation [38]. No previous studies have compared these groups in physiological stress pre- and post-CBT. 

### The Present Study

The purposes of this study were to explore racial/ethnic differences in biomarkers of stress among smokers enrolled in a behavioral intervention study, and the association between race/ethnicity and smoking relapse. Treatment-seeking African American and white smokers received group CBT plus transdermal nicotine patch (TNP) therapy. They also provided saliva samples for analysis of cortisol levels at baseline, end-of-treatment [EOT], and one-month follow-up. It was hypothesized that African Americans would demonstrate attenuated cortisol levels relative to whites, and that African Americans would be more likely to relapse at the one-month follow-up. 

## 2. Materials and Methods

### 2.1. Study Design

This pilot study was a 2 (racial group: African American or white) x 3 [time: baseline, EOT, and one month follow-up] quasi-experimental design. Participants received eight sessions of group CBT plus eight weeks of TNP. They also completed saliva collections for cortisol analysis at each time point. An a priori sample size calculation indicated that 82 participants would yield. 80 power to detect a statistically significant change in slopes using linear regression examining the difference between slopes for two groups.

### 2.2. Participants

Adult smokers were recruited from the community using newspaper, radio, and internet ads, flyers, word-of-mouth, and through community partnerships. Inclusion criteria were self-identification as black race and American ethnicity or non-Hispanic white; current smoker of at least five cigarettes/day; ages 18–65; able to read 5th grade English; permanent contact information; access to transportation; an expired carbon monoxide (CO) level of ≥ 8 ppm; and interest in quitting smoking. Respondents were excluded if they were receiving any tobacco cessation treatment, pregnant, breastfeeding, or reported contraindications for nicotine patches. 

### 2.3. Procedures

The study was approved by the University of Miami Institutional Review Board. Respondents were screened by telephone, and those who met the eligibility criteria were scheduled for study orientation. Those who attended orientation were enrolled in the study, provided written informed consent, and completed the baseline assessment. CBT groups were led by doctoral-level psychologists and trained para-professionals with backgrounds in behavioral sciences or public health. CBT consisted of eight sessions over four consecutive weeks. Four saliva samples per day were collected at baseline, the EOT, and at the one-month follow-up. Participants received incentives for completing each assessment ($20.00 at baseline and the EOT, and $30.00 at the one-month follow-up).

### 2.4. Intervention

The intervention was comprised of two components, group CBT and pharmacotherapy. Group CBT followed an established protocol with demonstrated efficacy in diverse smokers [36,39], including African Americans [37]. The content delivered psychoeducation on nicotine addiction, the symptoms and time course of nicotine withdrawal, identification and management of “high risk” situations, cognitive and behavioral coping responses, stress management, weight control, seeking social support, behavioral contracting, and relapse-prevention strategies. Participants also received eight weeks of TNP according to manufacturer guidelines. 

### 2.5. Measures

#### 2.5.1. Demographics

Participants reported age, sex, education (less than high school; high school; business or technical training; some college, college degree), income (under $10,000; $10,001 to $20,000; $20,001 to $40,000; > $40,000), and marital status (single; married/separated/divorced/widowed).

#### 2.5.2. Nicotine Dependence

Participants completed the Fagerström Test for Nicotine Dependence [40], a well-established measure of physical dependence to nicotine. Items included time to first cigarette, the hardest cigarette to give up, difficulty refraining, and smoking while ill (range 0–10, alpha = 0.75).

#### 2.5.3. Sleep

Because sleep-wake and light-dark cycles affect cortisol release [41,42], participants reported their average number of hours of sleep per day in the past month and time of day they usually go to sleep for full rest (i.e., not a nap). The time of day of usual bedtime was coded as overnight (8:00 pm or later) or during the day (5:00 pm or later). 

#### 2.5.4. Physiological Stress

Saliva cortisol measured physiological stress. Samples were collected upon waking, 30 minutes after waking, 4:00 pm, and at 6:30 pm at baseline, the EOT, and at the one-month follow-up. Among relatively healthy adults, cortisol has a diurnal cycle, peaking upon awakening and steadily decreasing over the day. Thus, we collected samples during a standardized period to control for diurnal and circadian rhythms. We used standard cortisol collection instructions (e.g., do not to eat/drink/brush teeth/smoke within 60 minutes of collection; refrain from vigorous exercise, and alcohol within 12 hours of collection, etc.). Participants saturated the Salimetrics oral swab (without chewing) with saliva for two minutes, and then immediately placed it in the centrifuge tube. Participants returned frozen samples to the lab on the day of collection, and were provided with cool packs to keep the samples cold if immediate freezing was not possible. The last sample of the day was collected in the lab. Saliva samples were stored in −80 °C lab freezers. Cortisol was measured in duplicate using standard radioimmunoassay procedures in micrograms of lead per deciliter of blood (μg/dL). 

#### 2.5.5. Smoking Relapse

Smoking status at session 1 (target quit day), the EOT, and at the one-month follow-up was confirmed biochemically using breath Vitalograph monitor carbon monoxide (CO) readings of at least 5 parts per million (ppm) distinguishing smokers from nonsmokers [43]. Smoking relapse was defined as confirmed abstinence at session 1, and a return to smoking (i.e., self-reported smoking on three consecutive days or one day on two consecutive weeks) [44] at the EOT or the one-month follow-up.

### 2.6. Statistical Analyses

**Figure 1:**
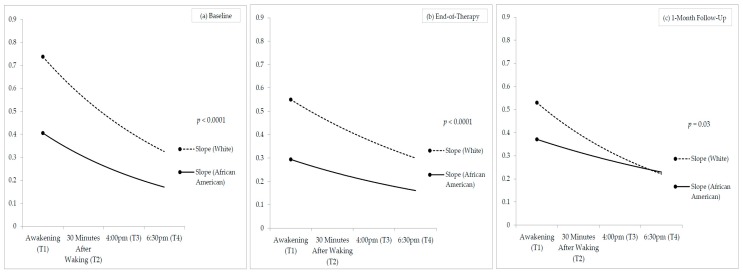
Cortisol Concentrations by Race/Ethnicity.

Descriptive analyses were conducted on demographic variables, nicotine dependence, and sleep. Salivary cortisol data were log-transformed and analyzed in two ways. First, we computed mean salivary cortisol levels over the course of the day (the 4 collections) at baseline, the EOT, and at the one-month follow-up. Repeated measures analyses of variance tested the effects of time of day, race/ethnicity, and their interactions on cortisol levels. Models controlled for income, education (continuous variables), and smoking status. Regression coefficient analyses (RCA) [45] were conducted to extract individual cortisol slopes at each time point for each participant (Figure 1). RCA is an increasingly applied method of analyzing within-subjects data, and allows estimation of regression coefficients individually for each participant. Specifically, the criterion variable (saliva cortisol concentration) was regressed on the predictor variable (time of collection) for each person. The extracted values for slopes and intercept were compared by race/ethnicity. Analysis of smoking status between the EOT and the follow-up was conducted with an intent-to-treat (ITT) approach, in which missing data was considered relapsed. Smoking relapse was coded as binary (1 = relapsed, 0 = abstinent). Multivariate logistic regression models examined the odds of smoking relapse at the one-month follow-up by race/ethnicity, while controlling for (1) demographic covariates and (2) demographic covariates and baseline cortisol slope. There was no evidence of significant multicollinearity. Alpha was set at *p* = 0.05, and analyses were conducted using SPSS version 24 and 25 (IBM, Armonk, NY, USA). 

## 3. Results

### 3.1. Participant Characteristics by Race/Ethnicity

A total of 115 individuals (n = 72 (63%) African Americans, n = 43 (37%) non-Hispanic whites) enrolled in the study. Most participants completed the CBT intervention (84%), and the EOT (n = 92, 80%) and one-month follow-up (n = 102, 89%) assessments with no differences by race/ethnicity (*p*s > 0.05). As shown in Table 1, the sample was mostly unmarried (86%), middle aged (M = 48 years, SD = 10.38), and male (56%). Most participants completed at least a high school education (85%), and 55% reported an annual household income of under $10,000. Compared to whites, African Americans reported lower levels of education (*p* < 0.01) and income (*p* = 0.04). Participants averaged 19 (SD = 10.43) cigarettes per day for 28 years (SD = 11.73), and moderate nicotine dependence (M = 5.70, SD = 2.19), with no significant differences by race/ethnicity. Most participants reported sleeping on an overnight schedule (83%) for 6.74 (SD = 2.8) hours, again with no differences by race/ethnicity.

### 3.2. Physiological Stress Overall and by Race/Ethnicity

Controlling for covariates, there was a statistically significant effect of time on cortisol level at baseline, (F(3, 267) = 21.21, *p* < 0.0001). In the overall sample, cortisol concentrations demonstrated the expected patterns over the course of the day (the four collections) at each time point (Table 2). At baseline, there was an increase between the awakening and 30-minutes post-awakening cortisol levels, with the lowest levels at the afternoon and early evening collections. The interaction between time and race/ethnicity was not significant. Controlling for covariates, there was a statistically significant effect of time on cortisol level at the EOT, (F(3, 213) = 12.52, *p* < 0.0001). At the EOT, there was an increase between the awakening and 30-minutes post-awakening cortisol levels, with the lowest levels at the afternoon and early evening collections. Again, the interaction between time and race/ethnicity was not significant. At the one-month follow-up, controlling for covariates, there was a statistically significant effect of time on cortisol level at the one-month follow-up, (F(3, 183) = 27.74, *p* < 0.0001). The awakening and 30-minutes post-awakening cortisol levels were equivalent, and showed declines in the afternoon and early evening collections. There was also a significant interaction between time and race/ethnicity (F(3, 183) = 3.49, *p* < 0.02), such that African Americans exhibited significantly lower awakening cortisol levels compared to whites. 

Results also demonstrated racial/ethnic differences in mean cortisol concentration at each assessment point (Table 2). At baseline, African Americans exhibited lower cortisol levels compared to whites over the course of the day (F(1, 89) = 12.43, *p* = 0.0010). Attenuated cortisol levels were also found when examined at each of the four collection times (*p*s < 0.05). At the EOT, African Americans exhibited lower cortisol levels compared to whites over the course of the day (F(1, 71) = 13.71, *p* < 0.0001), and at each of the four collection times (*p*s < 0.05). At the one-month follow-up, cortisol levels over the course of the day did not differ significantly by race/ethnicity (F(1, 61) = 2.45, *p* = 0.12). However, African Americans exhibited significantly lower awakening cortisol compared to whites (*p* < 0.05).

Figure 1 illustrates racial/ethnic differences in cortisol slopes at baseline, the EOT, and the one-month follow-up. RCA analyses indicated that cortisol slopes were significantly flatter among African Americans relative to whites at baseline (F(1, 407) = 23.02, *p* < 0.0001), at the end-of-therapy (F(1, 328) = 35.02, *p* < 0.0001), and at the one-month follow-up (F(1, 294) = 4.59, *p* = 0.03).

### 3.3. Smoking Relapse

Most participants maintained abstinence successfully through the EOT (73%). Between session 1 and the end-of-therapy, relapse rates were greater among African Americans (31.3%) compared to whites (17.5%). Controlling for demographic covariates, African Americans who quit at session 1 were almost four times more likely to have relapsed at the EOT compared to their white counterparts (*p* = 0.03; Table 3). When baseline cortisol slope was added to the model, this association was reduced (*p* = 0.054), suggesting partial mediation. Between session 1 and the one-month follow-up, relapse rates were greater among African Americans (50%) compared to whites (29.3%). Multivariable analyses also demonstrated that African Americans who quit at session 1 were almost three times more likely to have relapsed at the one-month follow-up compared to their white counterparts (*p* = 0.03; Table 3). When the baseline cortisol slope was added to the model, African Americans were almost four times as likely to relapse relative to whites (*p* = 0.01).

## 4. Discussion

This pilot study was the first to test and demonstrate racial/ethnic differences in HPA axis functioning between African American and white treatment-seeking tobacco smokers. In the overall sample, cortisol levels followed the commonly observed diurnal rhythm at baseline, with an increase 30 minutes after waking followed by lower concentrations in the evenings. At the EOT, overall cortisol concentrations were attenuated, and were somewhat higher at the one-month follow-up assessment. When compared by race/ethnicity, different patterns in cortisol levels emerged. Compared to whites, cortisol levels were lower over the course of the day among African Americans at baseline and the EOT. At the one-month follow-up, cortisol levels over the course of the day did not differ by race/ethnicity, although awakening concentrations were lower among African Americans. In addition, cortisol slopes were flatter among African Africans compared to whites at each time point. Finally, African Americans were more likely than whites to relapse to smoking at both the EOT and the one-month follow-up.

These results support previous reports of differences in physiological stress processes between African American and white participants—yet, none have reported this relationship among treatment-seeking tobacco smokers. Several studies have found lower awakening cortisol concentrations and flatter slopes among African Americans compared to whites [46,47]. The stability of both lower levels and blunted cortisol declines at three different time points among African Americans relative to Whites is indicative of HPA dysregulation and chronic distress. That is, this pattern reflects hormonal dysregulation likely resulting from ongoing exposure to psychosocial stressors. Dysregulation of allostatic mediators (e.g., cortisol levels, diurnal rhythm) precede various systemic and behavioral changes, including substance use. This studies provides initial evidence that HPA functioning may differ among African American tobacco smokers, which is important given the disproportionate burden of tobacco use on health in this population, and the role of stress in maintaining this addiction.

At the one-month follow-up, the racial/ethnic differences in mean cortisol concentrations over the day narrowed, although cortisol slopes remained significantly flatter among African Americans. In both groups, cortisol levels were somewhat attenuated compared to baseline, which is consistent with research showing lower cortisol levels post smoking cessation [48]. Previous research, however, assessed HPA activity following acute smoking abstinence, and the current findings signal that cortisol concentrations assessed two months post the target quit day remain lower than baseline. Although African Americans in this sample showed somewhat greater cortisol levels at the one-month follow-up compared to the EOT, the concentrations essentially returned to baseline, which were still lower than whites. Moreover, cortisol slopes remained significantly lower among African Americans at the follow-up. This consistent blunted HPA response may be related to the lower awakening cortisol levels among African Americans. This is a potential problem because higher awakening cortisol concentrations are important for effective preparation for the day [49] and lower awakening cortisol is related to health risks [50]. The good news is that stress is a modifiable risk factor for both smoking maintenance and poor health. The CBT intervention may have helped to narrow the physiological stress gap by race/ethnicity within the month following the end-of-therapy, a possible association supported by previous research [38]. Randomized intervention trials testing the impact of cognitive behavioral stress management [51] or distress tolerance therapy on cortisol concentrations over time and in the context of tobacco disparities are warranted. 

Smoking relapse is a significant concern and is positively associated with stress. About 80% of quit attempts result in relapse within a month [52]. Previous research has found a positive association between declines in cortisol levels and smoking relapse during week 1 of a cessation attempts [53]. In addition, smokers who relapsed within one-month of quitting demonstrated attenuated cortisol responses to stress [32]. Previous research has found that African Americans tend to be less successful in maintaining abstinence compared to their white counterparts [1,2,3,4,5,54]. Relapse rates in the current CBT intervention were relatively low, yet consistent with previous reports, African Americans were significantly more likely to relapse. Adjusted relapse risk models including baseline cortisol slope reduced the association between race/ethnicity and smoking relapse at the end-of-therapy, suggesting a partial mediation effect. Specifically, the flatter cortisol slope among African Americans at session 1 helped explain why this group was more likely to relapse at the end of CBT. This extends previous research indicating that African American smokers may be disadvantaged by greater distress at the outset of cessation treatment [38]. Given that the relationship was not completely reduced and that baseline cortisol slope did not mediate the race/ethnicity–smoking relapse association at the one-month follow-up, additional research is needed to elucidate these findings.

Results of this study contribute to our understanding of factors contributing to tobacco-associated health disparities. The allostasis model suggests that chronic stress exposures confer a differential vulnerability to health problems among African Americans [26], and a biobehavioral stress model in the recent National Cancer Institute monograph on tobacco disparities described similar processes for racial/ethnic minority tobacco users [55]. Large epidemiological studies indicate that the association between allostatic load (i.e., negative effects of stressful experiences and attempts to adapt over time) and mortality is stronger among African Americans compared to whites, even after controlling for socioeconomic status [56]. This supports the weathering hypothesis, which suggests that stress has a greater propensity to “get under the skin” among African Americans relative to their white counterparts [10], perhaps due to more prolonged or deep exposure to specific stressors such as racial discrimination. Chronic activation over the lifespan can result in dysregulation (i.e., hyporesponsivity or hyperresponsivity) of the physiological stress response, and accelerated wear and tear on the body [57]. As such, the toll of cumulative stress in this population may predict morbidity and death [19,58,59], particularly among those at risk for tobacco-associated illnesses.

This study has important strengths, yet is not without limitations. First, this study contributes to the body of knowledge regarding physiological stress among smokers, and expands the existing knowledge with a focus on racial/ethnic differences. Second, there are no previous studies examining racial/ethnic differences in smoking relapse following gold standard treatment. Further, the focus on HPA axis functioning provides an objective indicator of stress among smokers as they undergo CBT and thereafter. Study limitations include the small sample size, self-selection of highly motivated participants, the lack of data on nicotine withdrawal, and the short-term follow-up. Because this was a pilot study, never-smoker case controls were not included, which may offer insight on whether racial/ethnic differences in stress biomarkers are moderated by smoking status. Offsetting these limitations, however, are the consistency of the findings with previous research in other areas of health, the longitudinal repeated measures design that enhanced statistical power to detect effects, and the biochemical verification of smoking status. Moreover, this exploratory study confirms the need for additional studies to replicate and extend the current findings. 

## 5. Conclusions

In conclusion, findings from this study underscore the need to understand stress mechanisms among African American smokers and in the context of health disparities. The observed pattern of racial differences in salivary cortisol levels is consistent with previous literature in other domains of health, and extend this work to indicate that treatment-seeking African American smokers may have lower cortisol levels at the start of a quit attempt, and demonstrate hyporesponsive HPA axis functioning throughout the process. That is, the propensity for environmental stressors to “get under the skin” of African Americans in general also applies to tobacco smokers, and has implications for racial differences in smoking relapse. Findings also highlight the importance of multilevel culturally specific intervention approaches to help address this modifiable risk factor.

## Figures and Tables

**Table 1:** Demographic Characteristics by Racial/Ethnic Group.

Mean (SD)	White	African American	*p*-Value
	(n = 43)	(n = 72)	
Age (years)	46.2 (13.2)	49.5 (8.2)	0.09
Cigarettes per day	20.19 (10.6)	17.6 (10.2)	0.1
Years of smoking	29.1 (12.9)	27.8 (11.0)	0.56
Nicotine dependence (FTND)	5.9 (2.2)	5.6 (2.2)	0.41
Hours of Sleep	6.89 (3.2)	6.0 (2.4)	0.09
**% (n)**			
**Sex**			0.51
Male	61% (26)	54% (39)	
Female	40% (17)	46% (33)	
**Total annual household income**			<0.01
Under $10,000	43% (18)	63% (45)	
$10,000 to $20,000	17% (7)	10% (16)	
$20,001 to $40,000	17% (7)	14% (10)	
> $40,000	26% (11)	1% (1)	
**Marital status**			0.41
Single	42% (18)	58% (42)	
Married	13% (9)	13% (9)	
Separated/Divorced/Widowed	37% (16)	29% (21)	
**Education**			0.04
Less than high school	16% (7)	21% (15)	
High school	26% (11)	38% (27)	
Business or technical training	2% (1)	10% (7)	
Some college, no degree	28% (12)	26% (19)	
College degree	28% (12)	6% (4)	
**Sleep**			
Overnight Bedtime	85% (37)	80% (58)	0.45
Day Bedtime	15% (6)	20% (14)	

Note. FTND = Fagerström Test for Nicotine Dependence.

**Table 2:** Salivary Cortisol Concentrations by Racial/Ethnic Group.

Baseline (Session 1)	Overall	White	African American	*p*-Value
		Mean μg/dL (SD)		
Awakening (T1)	0.45 (0.41)	0.66 (0.55)	0.37 (0.30)	0.01
30 Minutes After Waking (T2)	0.48 (0.60)	0.71 (0.97)	0.37 (0.29)	0.03
4:00 pm (T3)	0.26 (0.24)	0.39 (0.29)	0.21 (0.21)	0.04
6:30 pm (T4)	0.21 (0.23)	0.31 (0.30)	0.17 (0.19)	0.01
End-of-Therapy				
Awakening (T1)	0.36 (0.31)	0.51 (0.40)	0.27 (0.18)	< 0.01
30 Minutes After Waking (T2)	0.38 (0.28)	0.49 (0.35)	0.31 (0.20)	< 0.01
4:00 pm (T3)	0.24 (0.28)	0.39 (0.40)	0.16 (0.12)	< 0.01
6:30 pm (T4)	0.21 (0.29)	0.24 (0.40)	0.17 (0.19)	0.04
1-Month Follow-up				
Awakening (T1)	0.40 (0.22)	0.48 (0.25)	0.35 (0.19)	0.04
30 Minutes After Waking (T2)	0.41 (0.25)	0.47 (0.28)	0.36 (0.21)	0.07
4:00 pm (T3)	0.27 (0.20)	0.28 (0.20)	0.26 (0.20)	0.99
6:30 pm (T4)	0.22 (0.16)	0.22 (0.16)	0.23 (0.16)	0.79

Note: T1 = first collection; T2 = second collection; T3 = third collection; T4 = fourth collection; μg/dL = micrograms of lead per deciliter of blood.

**Table 3:** Smoking Relapse by Racial/Ethnic Group.

Characteristic	Session 1 to EOT (*n* = 107)	Session 1 to EOT	Session 1 to 1-Month Follow-Up (*n* = 109)	Session 1 to 1-Month Follow-Up
	AOR ^1^ (95% CI)	AOR ^2^ (95% CI)	AOR ^1^ (95% CI)	AOR ^2^ (95% CI)
Race/Ethnicity				
White	Ref	Ref	Ref	Ref
African American	3.89 (1.10–13.78)	3.60 (0.98–13.24)	2.73 (1.08–6.91)	3.81 (1.36–10.64)
Education	1.11 (0.79–1.55)	1.10 (0.77–1.58)	1.17 (0.87–1.56)	1.20 (0.87–1.65)
Household Income	1.17 (0.91–1.51)	1.18 (0.87–1.60)	1.02 (0.81–1.27)	1.09 (0.83–1.42)
Baseline Cortisol Slope		0.54 (0.60–4.87)		0.97 (0.12–7.91)

Note. AOR ^1^ = Adjusted odds-ratio controlling for education and income; AOR ^2^ Adjusted odds-ratio controlling for income, education and baseline cortisol slope; EOT = end-of therapy.
